# Reproductive fitness and genetic risk of psychiatric disorders in the general population

**DOI:** 10.1038/ncomms15833

**Published:** 2017-06-13

**Authors:** Niamh Mullins, Andrés Ingason, Heather Porter, Jack Euesden, Alexandra Gillett, Sigurgeir Ólafsson, Daniel F. Gudbjartsson, Cathryn M. Lewis, Engilbert Sigurdsson, Evald Saemundsen, Ólafur Ó Gudmundsson, Michael L. Frigge, Augustine Kong, Agnar Helgason, G. Bragi Walters, Omar Gustafsson, Hreinn Stefansson, Kari Stefansson

**Affiliations:** 1 deCODE genetics, 101 Reykjavik, Iceland; 2 MRC Social, Genetic and Developmental Psychiatry Centre, Institute of Psychiatry, Psychology and Neuroscience, King’s College London, London SE5 8AF, UK; 3 Integrative Epidemiology Unit, Oakfield House, University of Bristol, Bristol BS8 2EG, UK; 4 Faculty of Medicine, University of Iceland, 101 Reykjavik, Iceland; 5 Division of Genetics and Molecular Medicine, King’s College London, London SE1 9RT, UK; 6 Department of Psychiatry, Landspitali University Hospital, 101 Reykjavik, Iceland; 7 The State Diagnostic and Counselling Centre, 200 Kópavogur, Iceland; 8 Department of Anthropology, University of Iceland, 101 Reykjavik, Iceland

## Abstract

The persistence of common, heritable psychiatric disorders that reduce reproductive fitness is an evolutionary paradox. Here, we investigate the selection pressures on sequence variants that predispose to schizophrenia, autism, bipolar disorder, major depression and attention deficit hyperactivity disorder (ADHD) using genomic data from 150,656 Icelanders, excluding those diagnosed with these psychiatric diseases. Polygenic risk of autism and ADHD is associated with number of children. Higher polygenic risk of autism is associated with fewer children and older age at first child whereas higher polygenic risk of ADHD is associated with having more children. We find no evidence for a selective advantage of a high polygenic risk of schizophrenia or bipolar disorder. Rare copy-number variants conferring moderate to high risk of psychiatric illness are associated with having fewer children and are under stronger negative selection pressure than common sequence variants.

## Abstract (web summary)

Why genetic variants that confer risk for psychiatric disorders persist in the genome is an evolutionary conundrum. Here, Mullins *et al*. report association of polygenic risk for autism with having fewer children and polygenic risk for ADHD with higher reproductive fitness.

Psychiatric disorders present a paradox which has long puzzled researchers. Mental illness usually begins in early reproductive age and those affected have fewer children than those unaffected by severe mental illness^1^,^2^. This, along with the substantial heritability and prevalence of psychiatric disorders, raises the question of why variants that increase the risk of such diseases have not been purged from the gene pool^3^,^4^. The reasons by which susceptibility alleles for mental disorders persist in the population remain elusive, but three key mechanisms have been proposed. Mutation-selection balance postulates that selection against deleterious sequence variants is balanced by the continuous occurrence of new mutations^2^,^3^,^5^. Balancing selection suggests that variants that predispose to psychiatric disorders may be beneficial under some circumstances, compensating for the deleterious effects attributable to these disorders^2^,^3^,^5^. A third possibility is an accumulation of common variants with effects on psychiatric disorders that are individually too weak to be effectively targeted by negative selection in most human populations^6^.

Rare recurrent copy-number variants (CNVs) with large effects have been associated with schizophrenia, autism and bipolar disorder in a subset of patients^7^,^8^. Two such CNVs have been associated with decreased reproductive fitness in carriers in the general population, with male fitness being particularly affected^9^. The persistence of these CNVs in the gene pool is consistent with mutation-selection balance. However, psychiatric disorders also have a polygenic component, arising from the combined effect of many common risk variants, each with small effect^4^. Polygenic risk scoring is a method of summarizing an individual’s genetic liability for a trait, by weighting alleles according to effect sizes estimated in genome-wide association studies (GWAS)^10^. These effects are summed together into a polygenic risk score (PRS), that reflects the cumulative impact of many common variants on a phenotype.

Association between PRS for psychiatric disorders and having fewer children in undiagnosed individuals from the general population would indicate that common risk variants are subject to negative selection. Association between PRS and having more children in individuals unaffected by psychiatric disorders would support balancing selection. Lack of association between PRS and number of children would indicate that there is either no selection affecting these variants or weak selection pressure which currently cannot be detected. Here, we test these hypotheses using data from a sample of 150,656 Icelanders representing approximately half of the population. CNVs implicated in autism and schizophrenia are also tested for association with number of children in carriers in the general population, to compare the selection pressures on the common and rare components of the genetic basis of psychiatric disorders. Finally, we determine whether PRS for psychiatric disorders are associated with age at first child in the Icelandic population, to further dissect the link between parental age and risk of psychiatric disorders^11^,^12^.

Our data show that polygenic risk for autism is associated with fewer children and later age at first child, while polygenic risk for ADHD is associated with having more children. We find no evidence for a selective advantage of high polygenic risk for schizophrenia or bipolar disorder. Rare CNVs conferring moderate to high risk of psychiatric illness are associated with having fewer children and are under stronger negative selection pressure than common genetic variants.

## Results

### PRS and psychiatric disorders

**Figure 1:**
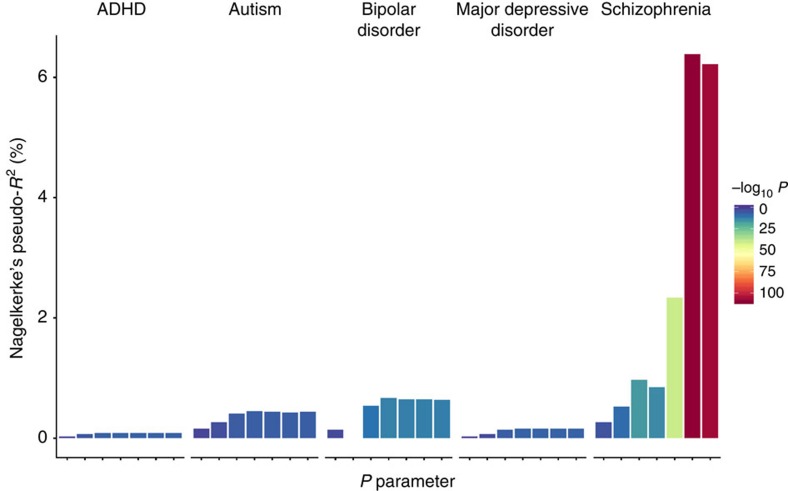
Polygenic risk scores for psychiatric disorders predict their corresponding disorder in the general population of Iceland. The *x* axis shows the seven *P*-value parameters (0.001, 0.003, 0.01, 0.03, 0.1, 0.3 and 1.0) used to weight SNPs from the discovery GWAS plotted left to right. The *y* axis indicates the Nagelkerke’s pseudo-*R*^2^ measure of variance explained.

PRS for five psychiatric disorders were generated for each genotyped individual using the results of independent GWAS on schizophrenia, bipolar disorder, autism, attention deficit hyperactivity disorder (ADHD) and major depression, available online from the Psychiatric Genomics Consortium (https://pgc.unc.edu/)^13^,^14^,^15^,^16^,^17^. We first tested the predictive power of each PRS for their corresponding disorder within the Icelandic sample. All scores were significantly associated with their matching disorder (Fig. 1). The maximum variance explained was 6.4% for schizophrenia (*P*=2.1 × 10^−109^). Other PRS explained up to 0.6% of the variance for the corresponding diseases (Fig. 1).

### PRS and number of children

We used a subset of 93,720 genotyped subjects, aged at least 45 years and without a diagnosis of a psychiatric disorder, to test the association of each PRS with the number of children born to each individual. There was a negative association between the PRS for autism and number of children (*β*=−0.25, *P*=0.002) (Table 1), while higher PRS for ADHD was associated with having more children (*β*=0.15, *P*=0.002) (Table 1). Other PRS were not associated with number of children after Bonferroni correction for multiple tests (Table 1, Supplementary Figs 1–5). The quadratic effects of the PRS were also examined, to test whether very high or low PRS may be associated with number of children, but these results were non-significant. Furthermore, there were no associations between variance in number of children and deciles of PRS in the total sample, males or females, after multiple testing correction (Supplementary Figs 6–10).

### Neuropsychiatric CNVs and number of children

In a subset of patients with schizophrenia or autism, CNVs are likely to be the strongest individual factors contributing to the pathogenesis of the disorder. Eleven CNVs conferring risk of schizophrenia or autism (‘neuropsychiatric CNVs’) were tested for association with number of children, excluding individuals with autism, schizophrenia, bipolar disorder and intellectual disability^7^,^8^. Collectively, carriers of a neuropsychiatric CNV (*N*=469) had significantly fewer children than non-carriers (*N*=91,987) (*β*=−0.279, *P*=0.0001), with a greater reduction in males than females (Table 2). After correction for multiple comparisons, the 16p11.2 deletion was individually associated with having fewer children (Table 2).

### Selection pressures on PRS versus neuropsychiatric CNVs

Individuals in the top 1% of schizophrenia PRS have an odds ratio of 9.5 (95% CI 6.8−13.3) of developing the disorder. While this risk is of a magnitude similar to that conferred by a single neuropsychiatric CNV, individuals with high PRS of schizophrenia have the same number of children as the rest of the population (*β*=0.054, *P*=0.29). However, they have a greater variance in number of children (*β*=1.112, *P*=0.02). The same comparison was performed for individuals in the top 1% of PRS for autism and bipolar disorder. The difference in number of children was not statistically significant in the former (*β*=−0.085, *P*=0.095, OR for autism=2.4) while those in the top 1% of bipolar disorder PRS were found to have fewer children than the rest of the population (*β*=−0.148, *P*=0.0037, OR for bipolar disorder=1.6).

### PRS and age at first child

PRS for autism is associated with later age at first child in the total sample (*β*=0.97, *P*=0.0004) (Table 3). PRS for ADHD is associated with younger age at first child for both sexes (*β*=−0.59, *P*=0.0003) and PRS for MDD is associated with younger age at first child in females (*β*=−0.56, *P*=0.00009) (Table 3). Quadratic effects of PRS were not found to be associated with age at first child.

## Discussion

Here we have investigated selection pressures acting on sequence variants conferring risk of psychiatric disorders in recent generations of Icelanders by testing whether PRSs and neuropsychiatric CNVs are associated with number of children in a large population sample that excludes patients diagnosed with the psychiatric diseases. PRS for autism was found to be associated with having fewer children, indicating that common variants that are thousands of years old are currently subject to weak negative selection pressure.

Balancing selection as an explanation for the persistence of these variants means that variants that increase the risk of psychiatric disorders may persist if their negative effects on fitness in affected carriers are offset by benefits in individuals who carry the variants but do not develop the disorders^3^. PRS for schizophrenia and bipolar disorder have been found to predict creativity in the general population of Iceland^18^. However, creative individuals in Iceland have fewer children than population controls^18^. Based on these results, along with the lack of association between these PRS and number of children found here, we conclude that there is no evidence for a selective advantage that maintains common variants associated with schizophrenia or bipolar disorder. While greater PRS for ADHD is associated with having more children in the Icelandic population, we do not interpret this as support for balancing selection. This is because individuals with ADHD have more children than average in Iceland and in other populations^19^.

We recognize that a Bonferroni correction may be over-conservative for this analysis, as one and the same variant may predispose to several psychiatric disorders, meaning that the PRS are not totally independent^20^,^21^. Furthermore, several of these PRS currently only explain a small amount of variance in risk of the disorders themselves (Fig. 1) and therefore may be underpowered to detect associations with number of children, even in this large population sample. As GWAS with greater statistical power are conducted on psychiatric disorders and their polygenic component can be estimated with greater accuracy, other associations with reproductive fitness may be uncovered. One limitation of the current study is that selection pressures in the modern environment may not be the same as those that acted on our ancestors. For example, in recent times there have been far-reaching cultural changes in factors such as education and the use of contraception, which have led to a postponement of having a first child and a reduction in number of children in many human populations. In our data, subjects diagnosed with psychiatric disorders were excluded. However, some unidentified patients in the sample could obscure evidence for balancing selection.

Here, neuropsychiatric CNVs implicated in schizophrenia, autism and bipolar disorder are associated with fewer children, particularly among males. These CNVs are rare and generally have large effects^7^,^8^. Several have been associated with lower IQ, cognitive deficits and other physical abnormalities, that may have a negative impact on reproductive fitness in population controls who have not been diagnosed with psychotic or neurodevelopmental disorders^9^,^22^. D*e novo* CNVs have been implicated in cases of schizophrenia, autism and bipolar disorder which supports mutation-selection balance. A previous study has also shown that there is a strong selection against schizophrenia-associated CNVs, such that these variants persist in the population for only a few generations after they arise^23^.

Finally, age at first child is of particular interest as Western society has experienced a rapid postponement of parenthood^24^, which has been associated with reduction in polygenic score for educational attainment^25^. Delayed fatherhood has been linked to risk of psychiatric disorders, widely assumed to be caused by the accumulation of *de novo* mutations in the spermatogonial stem cells of older males^26^,^27^. However, recent population genetic modelling suggests that these mutations are unlikely to explain much of the risk and a weak correlation between age at first child and genetic liability to psychiatric illness could account for the observed incidence of the disorders in the children of older fathers^11^. In accordance with this hypothesis, PRS for autism was positively associated with later age at first child in the Icelandic population, providing an alternative explanation of age-related mutations.

In summary, our results show that common sequence variants conferring risk of autism and ADHD are currently under weak selection in the general population of Iceland. However, rare CNVs that also impact cognition are under stronger selection pressure, consistent with mutation-selection balance. The hypothesis that a selective advantage accounts for the prevalence of sequence variants conferring risk of schizophrenia and bipolar disorder is unproven, but rather this empirical evidence suggests that common sequence variants largely escape selection as their individual effect sizes are weak.

## Methods

### Subjects

The study was approved by the National Bioethics Committee of Iceland and the Icelandic Data Protection Authority. Samples are from a population genetic biobank of 150,656 Icelanders established by deCODE genetics. Reproductive fitness was defined as the number of children born to individuals over 45 years. Subjects born before 1968 with matching genotypic data (*N*=93,720) were identified from deCODE’s nation-wide genealogy database. This contains information on year of birth, county of birth and numbers of children of Icelanders. Diagnoses of schizophrenia and bipolar disorder were assigned according to Research Diagnostic Criteria (RDC)^28^ through the use of the Schedule for Affective Disorders and Schizophrenia Lifetime Version (SADS-L)^29^. ADHD subjects were recruited from outpatient pediatric, child and adult psychiatry clinics in Iceland; ICD-10 diagnoses were made on the basis of standardized diagnostic assessments by experienced clinicians. Autism subjects were ascertained through the State Diagnostic Counseling Center and the Department of Child and Adolescent Psychiatry in Iceland and received ICD-10 diagnoses based on standardized diagnostic assessments by clinical specialists. Diagnoses of MDD were made by clinicians or based on the results of a semi-structured interview (CIDI), and were assigned according to DSM-III, ICD-9 or ICD-10 criteria. All diagnoses of recurrent depression were included (that is, mild, moderate and severe), but in the case of single episode depression mild cases were excluded. Characteristics of the sample are shown in Supplementary Table 1.

### Genotyping and imputation

Genotyping was performed on Illumina HumanHap (300, 370, 610, 1 M, 2.5 M) and IlluminaOmni (670, 1 M, 2.5 M, Express) SNP arrays^9^. BeadStudio (Illumina; version 2.0) was used to call genotypes, normalize signal intensity data and establish the log R ratio and B allele frequency at every SNP. Long-range haplotype phasing was achieved using an iterative algorithm, which phases a single proband at a time, given the available phasing information on all other individuals who share a long haplotype identically by state with the proband^30^. Given the large proportion of the Icelandic population that has been chip-typed, accurate genome-wide long-range phasing is possible for all chip-typed Icelanders. For long-range phased haplotype association analysis, the genome was then partitioned into non-overlapping fixed 0.3 cm bins. Within each bin, the haplotype diversity was consistent with the combination of all chip-typed markers in the bin. The whole genomes of 8,453 Icelanders were sequenced using Illumina technology to a mean depth of at least × 10 (median × 32). SNPs and indels were identified and genotypes called using join calling with the Genome Analysis Toolkit Haplotype Caller (GATK version 3.3.0) (ref. 31). The error rate of genotype calls made solely on the basis of next generation sequence data decreases as a function of sequencing depth. Taking advantage of the fact that all the sequenced individuals had also been chip-typed and long-range phased, information about haplotype sharing was utilized to minimize the number of such errors. Thus, the genotype call in cases where sequence reads were ambiguous would be informed by comparison with sequence reads of other individuals sharing haplotypes with the individual in question at the ambiguous site. To improve genotype quality and to phase the sequencing genotypes an iterative algorithm based on the IMPUTE HMM model^32^ and using the long range phased haplotypes was employed^33^. The same principle was then used to impute the sequence variants identified in the 8,453 sequenced Icelanders into 150,656 Icelanders who had been genotyped with various Illumina SNP arrays and their genotypes phased using long-range phasing^33^.

### Polygenic risk scoring and CNV selection

We derived PRSs from GWAS summary results available online from the Psychiatric Genomics Consortium (https://pgc.unc.edu/) for ADHD, autism, bipolar disorder, major depression and schizophrenia^13^,^14^,^15^,^16^,^17^. The number of cases in these studies was 896, 3,303, 7,481, 9,240 and 35,476 respectively. The deCODE sample was not part of these analyses. To compute the PRSs we used approximately 630,000 autosomal markers from a framework set of markers used in long-range haplotype phasing. The framework markers have been selected on the basis of various quality criteria including high genotype yield, Hardy–Weinberg equilibrium and consistency of allele frequencies across different Illumina array types. We estimated the linkage disequilibrium between markers using Icelandic samples and adjusted for it using LDpred^34^ a recently proposed method. PRSs were calculated with seven different settings of the *P* parameter (corresponding roughly to the fraction of causal markers^34^): 0.001, 0.003, 0.01, 0.03, 0.1, 0.3 and 1.0. Eleven CNVs conferring risk of schizophrenia or autism (‘neuropsychiatric CNVs’) were selected from the most recent review on CNVs in schizophrenia^7^ and the most recent analysis of CNVs in autism^8^.

### Statistical analysis

PRSs were first tested for association with their corresponding psychiatric disorder in the Icelandic population (*N*=1,137, 692, 806, 3,246 and 631 cases for ADHD, autism, bipolar disorder, major depression and schizophrenia respectively). This was performed using logistic regression with five principal components as covariates. Models were compared against a null model including covariates only to calculate the Nagelkerke’s pseudo-*R*^2^ measure of variance explained. A linear mixed effects model was used to test the association of number of children with PRS for the five psychiatric disorders. Number of children was regressed on the PRS of interest, covarying for year of birth, sex and interaction between the two, birth county of last child or birth county of the parent, five principal components and sibship (to account for relatedness). For each respective disorder we chose the PRS calculated with a *P* parameter corresponding to a fraction of causal markers of 0.3 and modelled the correlation with its respective disorder and then recalibrated the PRS to have a mean of 0 and a unit increase corresponding to a doubling of risk for the disorder. All predictors were modelled as fixed effects apart from sibship which was random. This model was compared against a null model including the covariates only. To test the quadratic effects of the PRS, a PRS squared term was added and PRS was included in the null model. Sex-specific analyses were also conducted. Individuals diagnosed with each psychiatric disorder were excluded, although it may not be possible to identify every past case in a general population sample. Age at first child was tested for association with each PRS in the same manner. To examine the relationship between variance in number of children and PRSs, PRSs were split into deciles and number of children was adjusted for all covariates. A linear regression was used to test the association between deciles of PRS and residual number of children in the total sample, males and females. Neuropsychiatric CNVs were examined for association with number of children using the following covariates: year of birth, sex and interaction between the two, birth county of last child or birth county of the parent, five principal components and the random effect of sibship. Individuals with autism, schizophrenia, bipolar disorder and intellectual disability were excluded from the CNV analyses.

### Data availability

Data supporting the findings of this study are available within the article and its Supplementary Information files. Summary level data from the PGC GWAS used to calculate PRS in this study were obtained from the PGC Downloads website (https://www.med.unc.edu/pgc/results-and-downloads/).

## Additional information

**How to cite this article:** Mullins, N. *et al*. Reproductive fitness and genetic risk for psychiatric disorders in the general population. *Nat. Commun.* **8**, 15833 doi: 10.1038/ncomms15833 (2017).

**Publisher’s note:** Springer Nature remains neutral with regard to jurisdictional claims in published maps and institutional affiliations.

## Supplementary Material

Supplementary Information Supplementary Figures and Supplementary Tables.

## Figures and Tables

**Table 1:** Association between polygenic risk scores and number of children.

	**Total population**		**Males**		**Females**	
**Polygenic score**	***P*** **value**	**Beta (CI)**	***P*** **value**	**Beta (CI)**	***P*** **value**	**Beta (CI)**
ADHD	0.002	0.15 (0.05, 0.25)	0.170	0.09 (−0.04, 0.24)	0.002	0.20 (0.07, 0.33)
Autism	0.002	−0.25 (−0.41, −0.09)	0.003	−0.36 (−0.59, −0.12)	0.130	−0.16 (−0.38, 0.05)
Bipolar disorder	0.740	−0.005 (−0.03, 0.02)	0.500	0.01 (−0.02, −0.05)	0.310	−0.02 (−0.05, 0.01)
Major depression	0.170	0.04 (−0.01, 0.11)	0.650	0.02 (−0.07, 0.12)	0.094	0.07 (−0.01, 0.16)
Schizophrenia	0.160	0.006 (−0.002, 0.01)	0.530	0.004 (−0.008, 0.02)	0.170	0.007 (−0.003, 0.02)

In total, ten tests on number of children were performed; thus the significance threshold is *P*<0.005.

CI—95% confidence interval

**Table 2:** Association between neuropsychiatric CNVs and number of children.

**CNV**	**Odds ratio (autism/schizophrenia)**	**Carriers (male/female)**	**Non-carriers (male/female)**	**Beta (male/female)**	***P*** **value (male/female)**
All CNVs		201/268	42,826/49,161	−0.405/−0.184	0.00028/0.050
Autism CNVs		37/60	42,990/49,369	−1.263/−0.481	9.4E−07/0.014
Schizophrenia CNVs		189/256	42,838/49,173	−0.266/−0.115	0.021/0.23
16p11.2 del	inf/NA	12/12	43,015/49,417	−2.534/−1.59	1.8E−08/0.00025
1q21.1 del	NA/8.35	11/15	43,016/49,414	−0.741/−0.98	0.12/0.012
22q11.21 del	NA/inf	3	92,453	−1.421	0.11
16p11.2 dup	4.1/11.52	39	92,417	−0.326	0.19
15q11.2—13.1 dup	inf/13.20	4	92,452	−0.926	0.23
7q11.23 (WBS) dup	NA/11.35	3	92,453	−1.150	0.23
15q13.3 all del	inf/7.52	19	92,437	−0.343	0.34
16p13.1 dup	NA/2.30	103	92,353	−0.128	0.41
2p16.1 (NRXN1) del	5.6/9.01	11	92,445	−0.353	0.45
15q11.2 all del	NA/2.15	192	92,264	−0.058	0.61
1q21.1 dup	NA/3.45	38	92,418	0.049	0.85

Results are shown in males and females separately when there is a significant (*P*<0.05) association in either group. Counting all models fitted, 28 tests were performed; thus the significance threshold is *P*<0.0017. Odds ratios for autism and schizophrenia are from the literature^7^,^8^.

**Table 3:** Association between polygenic risk scores and age at first child.

	**Total sample**		**Males**		**Females**	
**Polygenic score**	***P*** **value**	**Beta (CI)**	***P*** **value**	**Beta (CI)**	***P*** **value**	**Beta (CI)**
ADHD	0.0003	−0.59 (−0.92, −0.26)	0.07	−0.48 (−0.99, −0.03)	0.0005	−0.71 (−1.12, −0.31)
Autism	0.0004	0.97 (0.43, 1.51)	0.03	0.91 (0.08, 1.76)	0.001	1.10 (0.45, 1.77)
Bipolar disorder	0.005	0.14 (0.04, 0.23)	0.18	0.10 (−0.04, 0.25)	0.007	0.16 (0.04 − 0.28)
Major depression	0.006	−0.31 (−0.54, −0.09)	0.71	−0.06 (−0.42, 0.28)	9.00E−05	−0.56 (−0.84, −0.28)
Schizophrenia	0.07	−0.02 (−0.05, 0.003)	0.81	−0.005 (−0.05, 0.04)	0.02	−0.04 (−0.07, −0.01)

In total, ten tests on age at first child were performed; thus the significance threshold is *P*<0.005.

CI—95% confidence interval
