# Supplementary material for: Reproductive fitness and genetic risk of psychiatric disorders in the general population
Source: Nat Commun. 2017 Jun 13;8:15833. doi: 10.1038/ncomms15833 (PMC5474730; doi:10.1038/ncomms15833)
Supplement: Supplementary Information — Supplementary Figures and Supplementary Tables. [file ncomms15833-s1.pdf]

Supplementary Table 1: Sample characteristics of genotyped individuals born before 1968 (n = 93,720)

Mean age (years) (s.d.)	68.91 (14.55)
Male (%)	43 606 (46.53)
Female (%)	50 114 (53.47)
Mean number of children (s.d.)	2.91 (1.60)
Mean age at first child (years) (s.d.)	24.09 (5.15)
Attention deficit hyperactivity disorder (%)	105 (0.11)
Autism (%)	145 (0.15)
Bipolar disorder (%)	653 (0.70)
Major depressive disorder (%)	2189 (2.33)
Schizophrenia (%)	463 (0.49)

s.d. - standard deviation

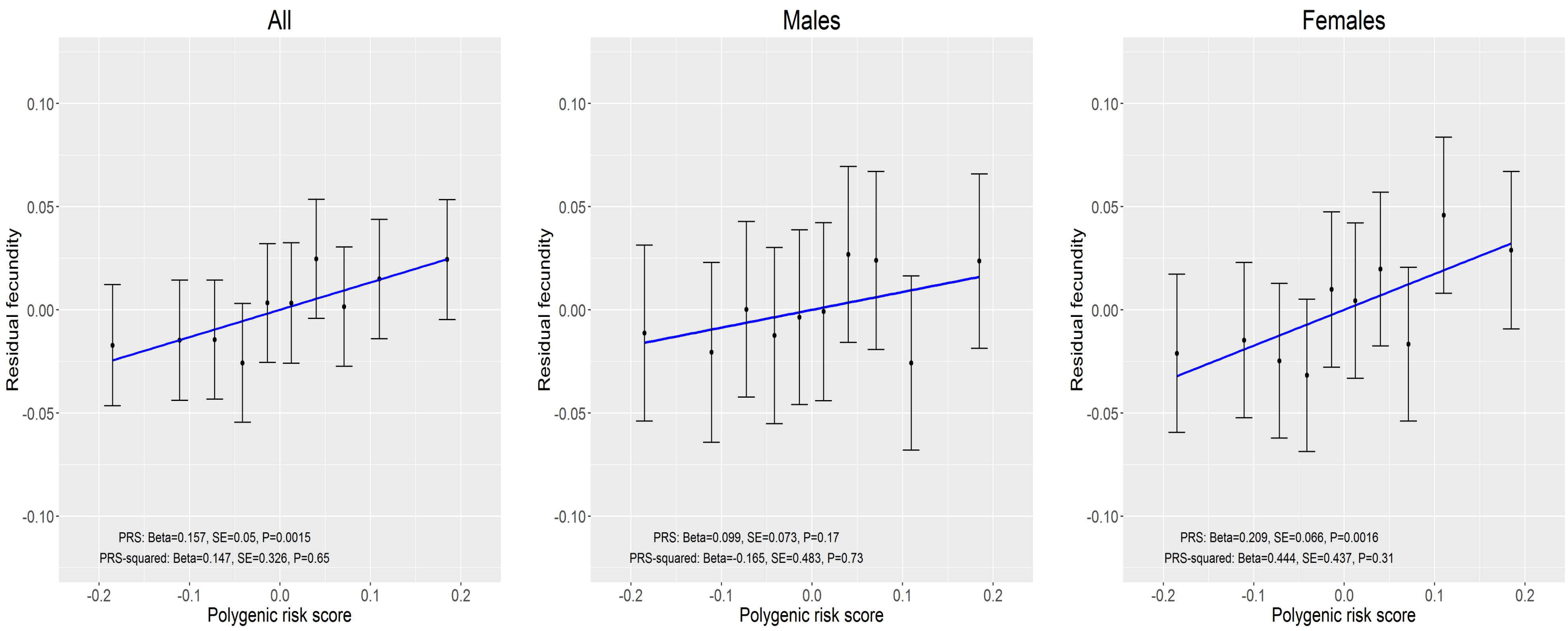

Supplementary Figure 1: ADHD polygenic risk score decile versus residual number of children in the total sample, males and females
Residual fecundity is number of children adjusted for year of birth, birth county of the last child, 5 principal components and sibship as a random effect. Polygenic risk scores were calculated using *P* value parameter 0.3 and recalibrated to have a mean of 0 and a unit increase corresponding to a doubling of risk for the disorder. The x-axis shows the mean polygenic risk score per decile. Affected patients are excluded.

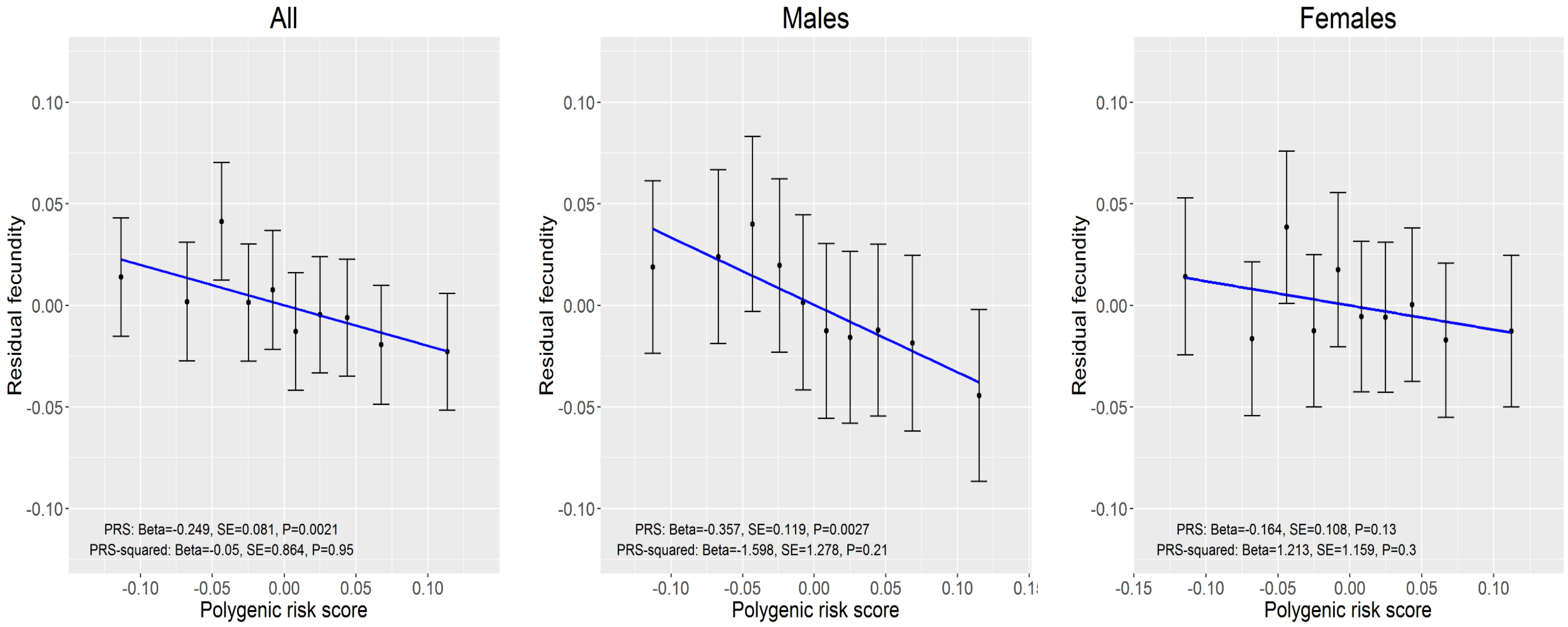

Supplementary Figure 2: Autism polygenic risk score decile versus residual number of children in the total sample, males and females
Residual fecundity is number of children adjusted for year of birth, birth county of the last child, 5 principal components and sibship as a random effect. Polygenic risk scores were calculated using *P* value parameter 0.3 and recalibrated to have a mean of 0 and a unit increase corresponding to a doubling of risk for the disorder. The x-axis shows the mean polygenic risk score per decile. Affected patients are excluded.

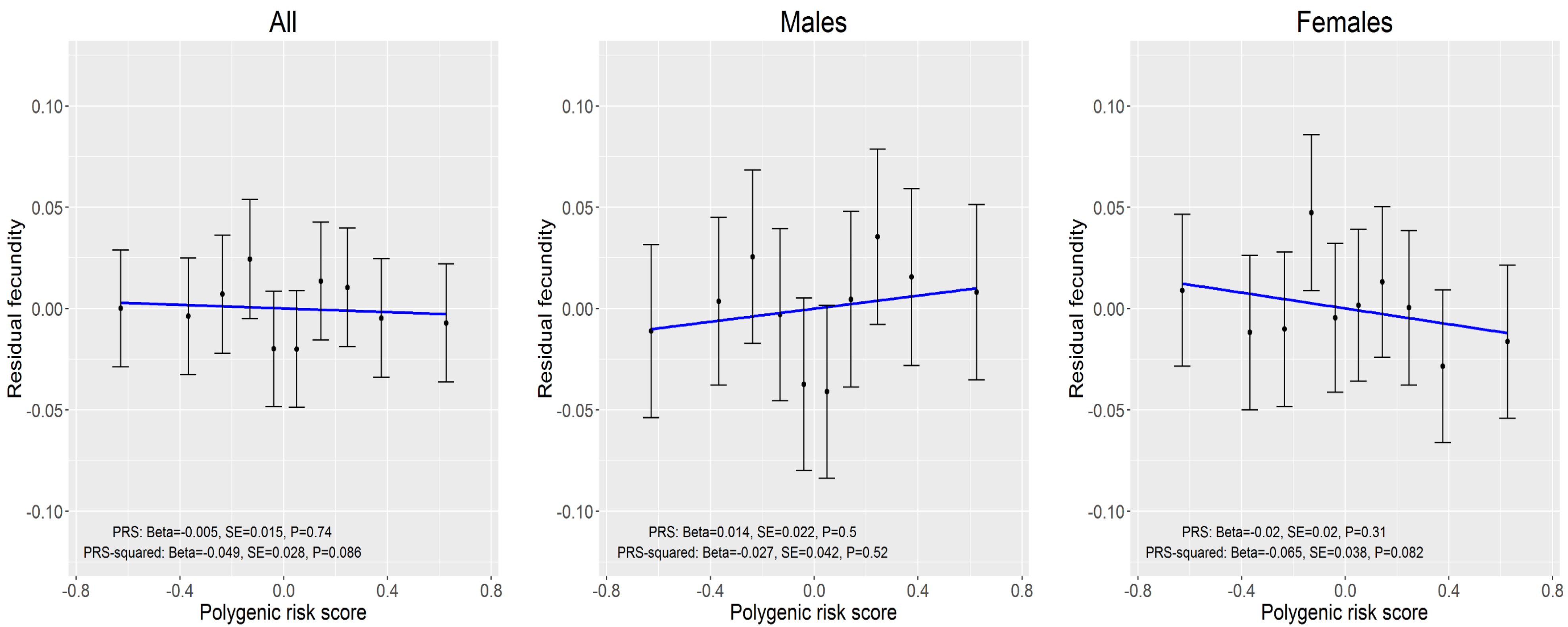

Supplementary Figure 3: Bipolar disorder polygenic risk score decile versus residual number of children in the total sample, males and females
Residual fecundity is number of children adjusted for year of birth, birth county of the last child, 5 principal components and sibship as a random effect. Polygenic risk scores were calculated using *P* value parameter 0.3 and recalibrated to have a mean of 0 and a unit increase corresponding to a doubling of risk for the disorder. The x-axis shows the mean polygenic risk score per decile. Affected patients are excluded.

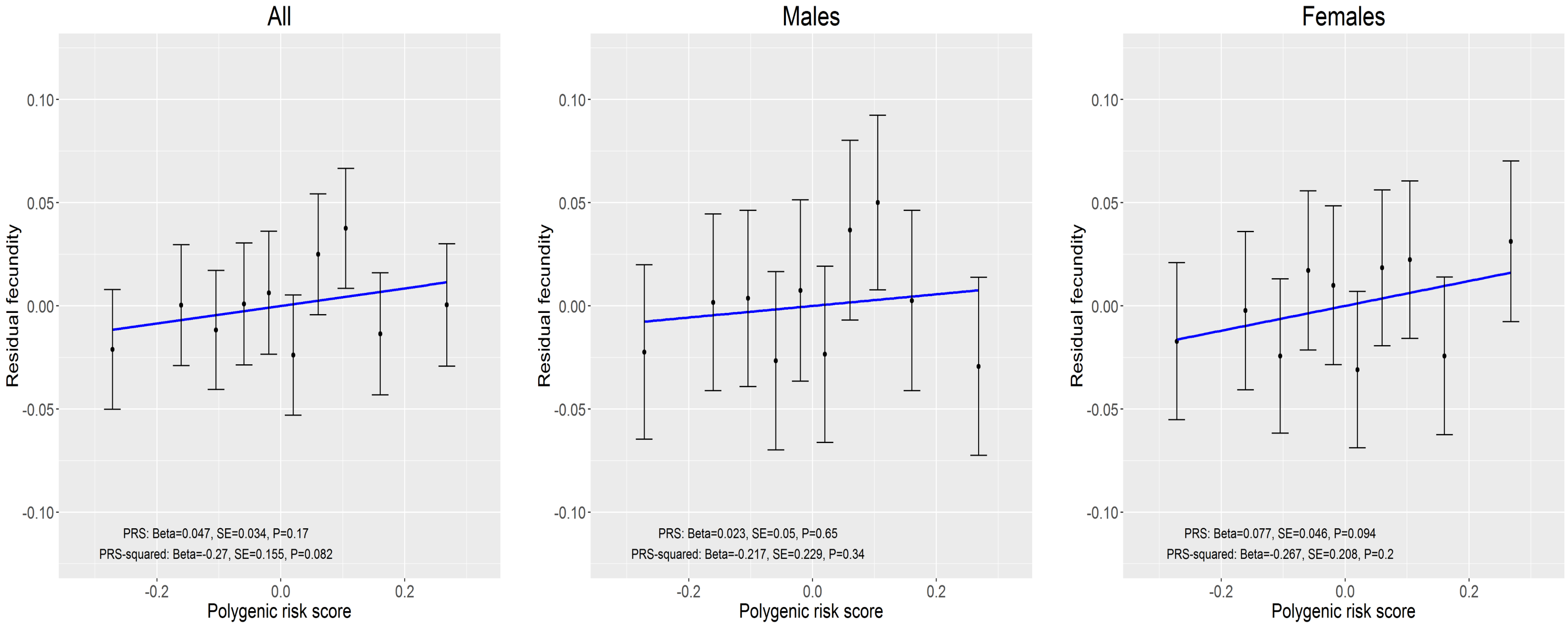

Supplementary Figure 4: Major depression polygenic risk score decile versus residual number of children in the total sample, males and females
Residual fecundity is number of children adjusted for year of birth, birth county of the last child, 5 principal components and sibship as a random effect. Polygenic risk scores were calculated using *P* value parameter 0.3 and recalibrated to have a mean of 0 and a unit increase corresponding to a doubling of risk for the disorder. The x-axis shows the mean polygenic risk score per decile. Affected patients are excluded.

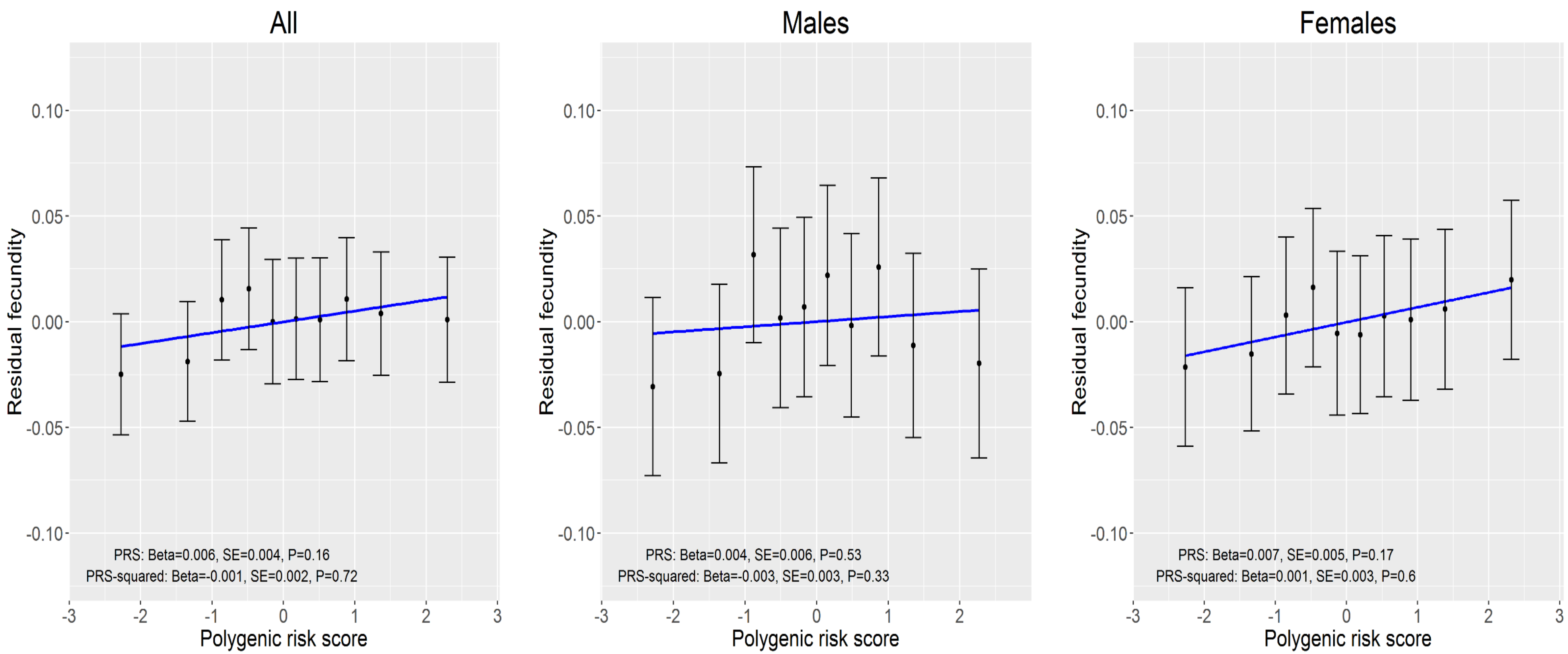

Supplementary Figure 5: Schizophrenia polygenic risk score decile versus residual number of children in the total sample, males and females
Residual fecundity is number of children adjusted for year of birth, birth county of the last child, 5 principal components and sibship as a random effect. Polygenic risk scores were calculated using *P* value parameter 0.3 and recalibrated to have a mean of 0 and a unit increase corresponding to a doubling of risk for the disorder. The x-axis shows the mean polygenic risk score per decile. Affected patients are excluded.

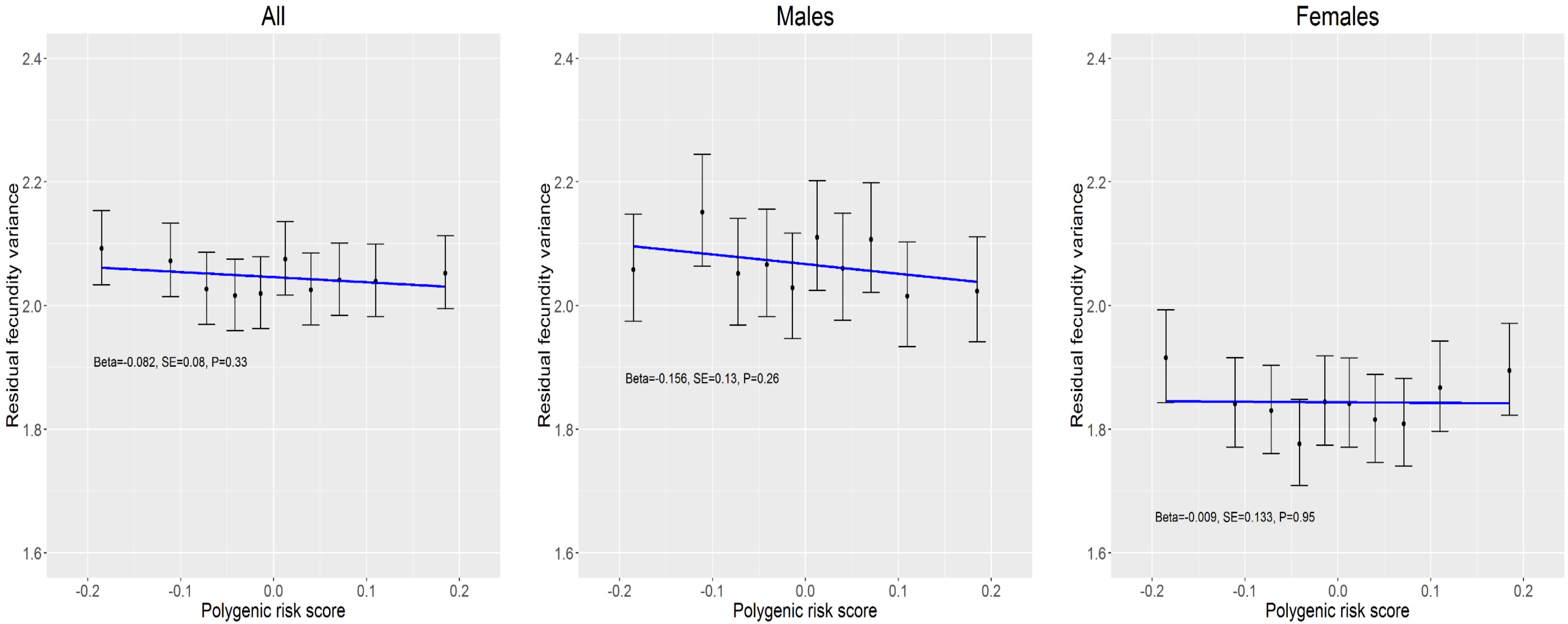

Supplementary Figure 6: ADHD polygenic risk score decile versus variance in residual number of children in the total sample, males and females
Residual fecundity is number of children adjusted for year of birth, birth county of the last child, 5 principal components and sibship as a random effect. Polygenic risk scores were calculated using *P* value parameter 0.3 and recalibrated to have a mean of 0 and a unit increase corresponding to a doubling of risk for the disorder. The x-axis shows the mean polygenic risk score per decile. Affected patients are excluded.

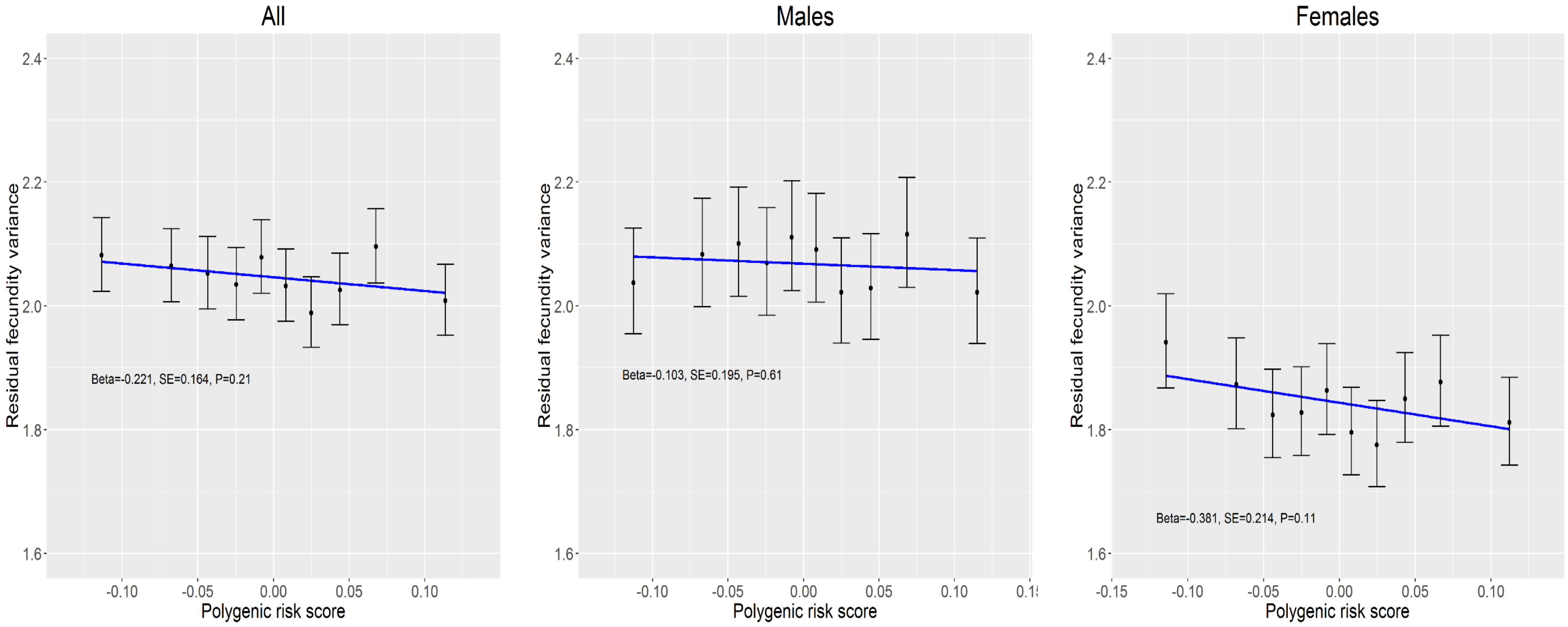

Supplementary Figure 7: Autism polygenic risk score decile versus variance in residual number of children in the total sample, males and females
Residual fecundity is number of children adjusted for year of birth, birth county of the last child, 5 principal components and sibship as a random effect. Polygenic risk scores were calculated using *P* value parameter 0.3 and recalibrated to have a mean of 0 and a unit increase corresponding to a doubling of risk for the disorder. The x-axis shows the mean polygenic risk score per decile. Affected patients are excluded.

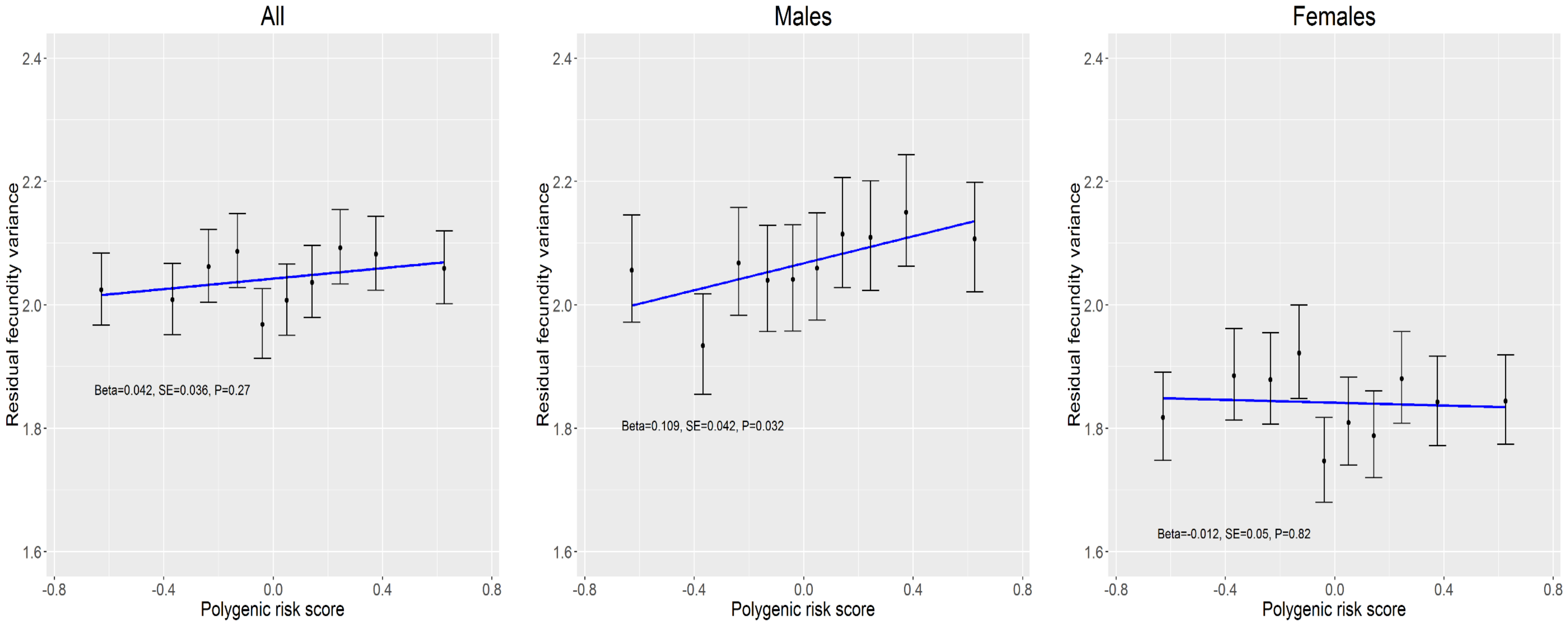

Supplementary Figure 8: Bipolar disorder polygenic risk score decile versus variance in residual number of children in the total sample, males and females
Residual fecundity is number of children adjusted for year of birth, birth county of the last child, 5 principal components and sibship as a random effect. Polygenic risk scores were calculated using *P* value parameter 0.3 and recalibrated to have a mean of 0 and a unit increase corresponding to a doubling of risk for the disorder. The x-axis shows the mean polygenic risk score per decile. Affected patients are excluded.

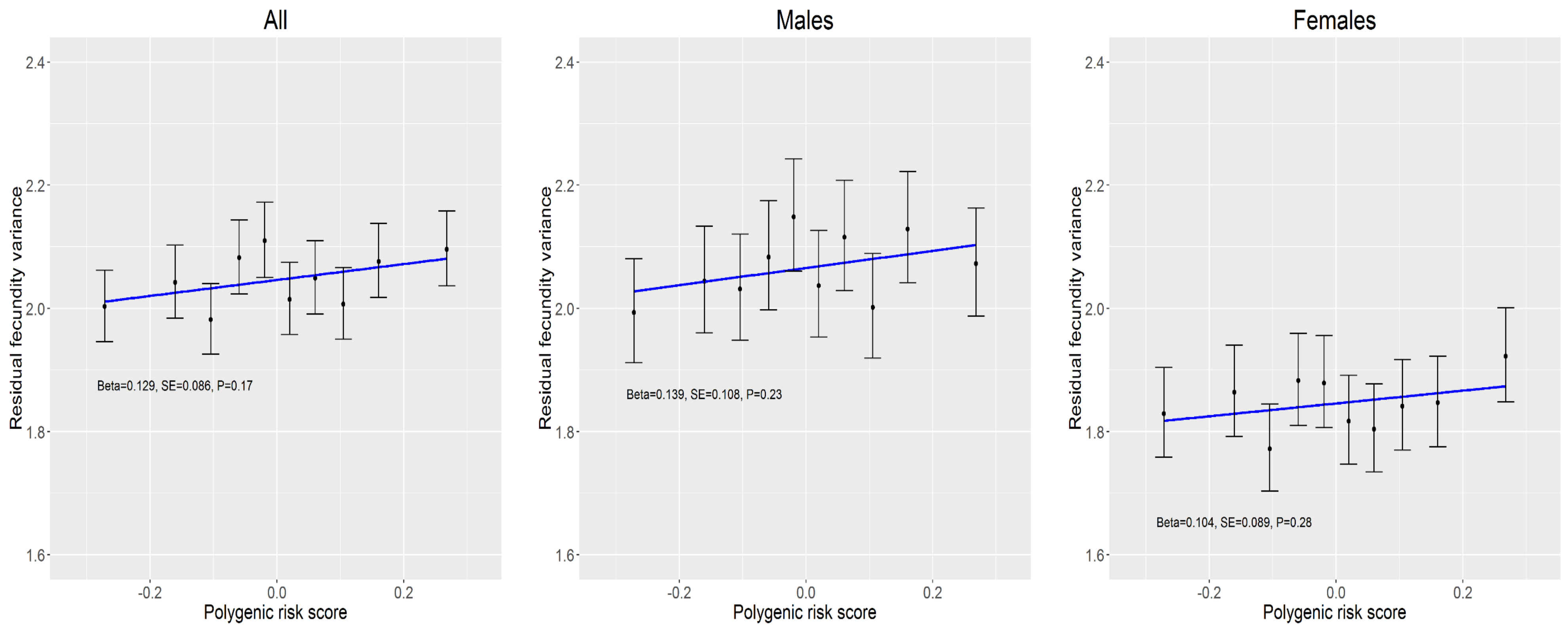

Supplementary Figure 9: Major depression polygenic risk score decile versus variance in residual number of children in the total sample, males and females
Residual fecundity is number of children adjusted for year of birth, birth county of the last child, 5 principal components and sibship as a random effect. Polygenic risk scores were calculated using *P* value parameter 0.3 and recalibrated to have a mean of 0 and a unit increase corresponding to a doubling of risk for the disorder. The x-axis shows the mean polygenic risk score per decile. Affected patients are excluded.

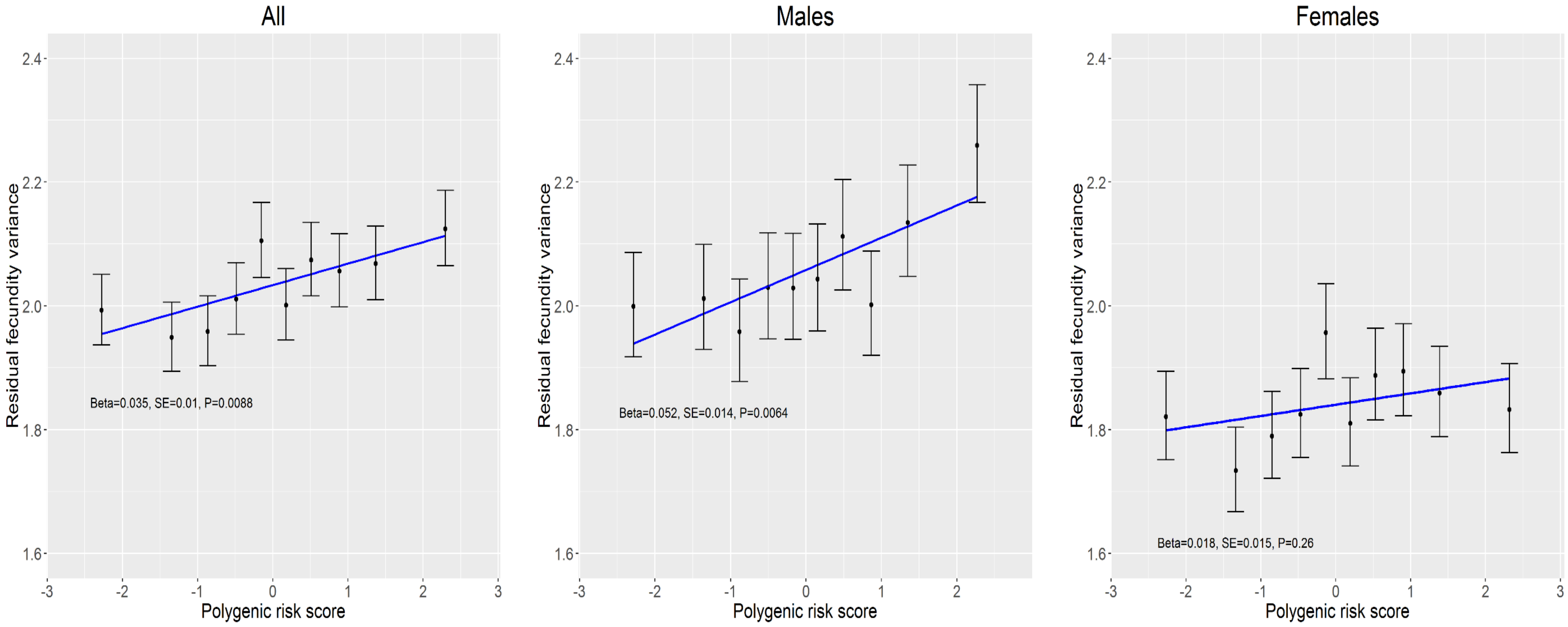

Supplementary Figure 10: Schizophrenia polygenic risk score decile versus variance in residual number of children in the total sample, males and females
Residual fecundity is number of children adjusted for year of birth, birth county of the last child, 5 principal components and sibship as a random effect. Polygenic risk scores were calculated using *P* value parameter 0.3 and recalibrated to have a mean of 0 and a unit increase corresponding to a doubling of risk for the disorder. The x-axis shows the mean polygenic risk score per decile. Affected patients are excluded.
